# Effects of Single Probiotic- and Combined Probiotic-Fermented Milk on Lipid Metabolism in Hyperlipidemic Rats

**DOI:** 10.3389/fmicb.2019.01312

**Published:** 2019-06-12

**Authors:** Yunchao Wa, Boxing Yin, Yong He, Wenbo Xi, Yingping Huang, Chunlei Wang, Feixiang Guo, Ruixia Gu

**Affiliations:** ^1^Jiangsu Key Laboratory of Dairy Biotechnology and Safety Control, Yangzhou University, Yangzhou, China; ^2^College of Animal Science and Technology, Yangzhou University, Yangzhou, China; ^3^Uni-President China Holdings Ltd., Shanghai, China; ^4^College of Food Science and Engineering, Yangzhou University, Yangzhou, China

**Keywords:** lipid metabolism, hyperlipidemic rats, probiotics, fermented milk, LXRs, AMPK, FXR

## Abstract

Previous studies have shown that probiotics have positive effects on hyperlipidemia by lowering the serum lipid concentration and improving the lipid profile. To explore the mechanism by which probiotic-fermented milk improves lipid metabolism, the transcription of genes regulated by liver X receptors (LXRs), 5′-AMP-activated protein kinase, and the farnesoid X receptor (FXR), which play integral roles in lipid metabolism, was investigated in hyperlipidemic rats. Compared with rats fed a high-fat diet, the administration of probiotic-fermented milk significantly lowered the levels of total cholesterol (TC) and total triglycerides (TG) in rat serum and viscera (*P* < 0.05) and significantly increased the level of total bile acid in the rat liver and small intestine (*P* < 0.05). The quantitative PCR results showed that the probiotics ameliorated the TC levels in the rats by activating the transcription of genes involved in the LXR axis, which promoted TC reverse transport and increased the conversion of TC to bile acids. The level of TG in the hyperlipidemic rats was ameliorated by the inhibition of the transcription of carbohydrate reaction element binding protein genes and activation of the transcription of PPARα genes. The regulation of lipid metabolism-related gene transcription by the single probiotic (*Lactobacillus rhamnosus* LV108)-fermented milk was more effective than that by the combined probiotic (*L. rhamnosus* LV108, *Lactobacillus casei* grx12, and *Lactobacillus fermentum* grx08)-fermented milk (*P* < 0.05).

## Introduction

Hyperlipidemia is a common cardiovascular disease posing a serious threat to human health. With the change in dietary structure, high-fat diets have become an important factor in hyperlipidemia, causing the incidence of hyperlipidemia to increase each year. However, other cardiovascular diseases, such as atherosclerosis, coronary heart disease, and hypertension, are also potential risks. Mann (1974) observed the hypocholesterolemic activity of fermented milk in a Maasai tribe in Kenya. Subsequently, animal and human models have been used to evaluate the effects of probiotic microorganisms on serum lipid levels, and probiotic benefits have been emphasized over the last 40 years. Accumulating studies have shown that well-established probiotics, prebiotics, and synbiotics possess hypocholesterolemic and other effects that modulate serum lipids in humans and animals (He and Shi, 2017). Currently, the mechanisms by which probiotics and their fermentation products reduce lipid levels mainly include the adhesion and absorption of living bacteria cells, the production of short chain fatty acids, the reduction of the reabsorption of bile acids, and the inhibition of lipoprotein lipase activity.

Recently, several studies have shown that liver X receptors (LXRs), 5′-AMP-activated protein kinase (AMPK), the farnesoid X receptor (FXR) and their downstream target genes play important roles in lipid metabolism (Tu et al., 2000; Geyeregger et al., 2006; Yao et al., 2016). LXRs, which are nuclear receptors activated by oxidized sterols, are widely expressed in the liver and small intestine and regulate some key genes in cholesterol metabolism (Zelcer and Tontonoz, 2006). The main function of LXRs includes limiting cholesterol accumulation in macrophages and other peripheral cell types by increasing the expression of the reverse cholesterol transporters ATP-binding cassette members A1 (ABCA1), G1 (ABCG1), and G5 (ABCG5), resulting in a program of reverse cholesterol transport (RCT) (Song et al., 1994; Willy et al., 1995; Tall et al., 2008). AMPK is a serine/threonine kinase widely found in eukaryotic cells that play an important role in maintaining the balance of cellular energy metabolism (Yao et al., 2016). Once activated, AMPK stimulates the activator of the oxide enzyme to activate receptor α (PPARα), upregulating the related target genes, promoting fatty acid oxidation to enhance the body’s catabolism, enhancing the β-oxidation of fatty acids, and promoting the production of ATP (Patel et al., 2014). AMPK can also inhibit the expression and transcriptional activity of sterol regulatory element binding protein (SREBP-1c) and carbohydrate reaction element binding protein (ChREBP), downregulating the transcription of related target genes and inhibiting lipid synthesis (Tu et al., 2000; Yoshikawa et al., 2001). Bile acids are endogenous FXR ligands expressed in the small intestine (Tu et al., 2000). FXRs can maintain the internal stability of bile acids by regulating the transcription of genes involved in bile acid metabolism (Tu et al., 2000). In addition, FXRs regulate the synthesis and transport of bile acids and prevent the excessive accumulation of bile acids in hepatocytes by inhibiting CYP7A1 transcription (Tu et al., 2000).

Recently, some studies have shown that probiotics can inhibit cholesterol uptake and increase cholesterol efflux by activating LXRs, eventually reducing the overall cholesterol level and potential risk of developing atherosclerosis (Huang et al., 2015). To the best of our knowledge, few studies have focused on the effects of probiotics on the multiple pathways of lipid metabolism or compared the effects of probiotics and combined probiotics on gene transcription in the lipid metabolism pathway. In this study, *Lactobacillus rhamnosus* LV108, *Lactobacillus casei* grx12, and *Lactobacillus fermentum* grx08 were isolated from human bodies at the Long Life Village in China (Chen, 2014). These three strains possess good acid resistance, artificial gastrointestinal tolerance, and bile salt tolerance *in vitro*. These three strains have been shown to improve the intestinal flora, antagonize pathogenic bacteria, modulate the immune response, possess anti-oxidation activity, and prevent tumors in animal models. Regarding blood lipid regulation, our previous study showed that *L. rhamnosus* LV108, *L. casei* grx12, and *L. fermentum* grx08 can reduce cholesterol *in vivo* and *in vitro* (Chen, 2014; Huang et al., 2013; Liu et al., 2016).

In this study, hyperlipidemic rats were used as a model to investigate the effects of fermented milk containing *L. rhamnosus* LV108, *L. casei* grx12, and *L. fermentum* grx08 on the transcription levels of lipid metabolism genes. Simultaneously, the effects of single probiotic-fermented milk and a mixture of milks fermented with three probiotics on hyperlipidemic rats were analyzed and compared.

## Materials and Methods

### Probiotics and Preparation of Milk Fermented With Probiotics

The starter cultures (*L. rhamnosus* LV108, *L. casei* grx12, and *L. fermentum* grx08) were provided by Jiangsu Province Key Laboratory of Dairy Processing and Safety, Yangzhou University. Each activated culture was inoculated into de Man, Rogosa, and Sharpe (MRS) medium (Haibo Biotechnology Co., Ltd., Qingdao, China) and incubated at 37°C for 12 h.

Skim milk (12%, w/w) was heat-treated at 95°C for 15 min and cooled to 37°C. The milk was fermented with *L. rhamnosus* LV108, *L. casei* grx12, or *L. fermentum* grx08 at 37°C for 20 h and stored at 4°C. The *L. rhamnosus* LV108-fermented milk was fermented with *L. rhamnosus* LV108, and the combined probiotic-fermented milk was a mixture of the three milks fermented with a single probiotic strain at a 1:1:1 (*w*:*w*:*w*) ratio. The number of viable bacteria in each milk fermented with a single probiotic strain and the combined probiotic-fermented milk was quantified to ensure that the number of viable bacteria was above 1 × 10^10^ cfu⋅mL^-1^. The two probiotic-fermented milks used in this study contained the same total viable count.

### Animals, Diet, and Experimental Design

Forty male Sprague-Dawley rats aged 5 weeks and weighing 140 ± 4.5 g were purchased from the Comparing Medical Center of Yang Zhou University (Jiangsu, China). The rats were individually housed in stainless steel cages in a pathogen-free room at a controlled temperature (22 ± 2°C) with a relative humidity of 55 ± 5% under a 12 h light–dark cycle. All animal procedures complied with the Guide for the Care and Use of Laboratory Animals and were approved by the Animal Care Committee of the Centers for Disease Control and Prevention (Jiangsu Province, China).

After 1 week of acclimation, the rats were randomly divided into four groups with 10 individuals per group. The groups included a control group (C), a high-fat model group (HF), a single probiotic (*L. rhamnosus* LV108)-fermented milk group (HFPB), and a combined probiotic-fermented milk (the mixture consisted of *L. rhamnosus* LV108-fermented milk, *L. casei* grx12-fermented milk, and *L. fermentum* grx08-fermented milk) group (HFPBS). Before the experiment, the rats in all groups, except for those in the C group, were fed a high-fat diet for 4 weeks, and their blood was collected to determine whether they exhibited hyperlipidemia. During the experiment, the rats in all groups, except for those in the C group, continued to be fed a high-fat diet.

All treatments were implemented by means of intragastric administration. The administered dose was determined according to the weight of the rats (1 mL100 g^-1^), and continuous intragastric administration was performed for 4 weeks. The C and HF groups were treated with normal saline, the HFPB group was treated with milk fermented with *L. rhamnosus* LV108 (total viable count > 1 × 10^10^ cfumL^-1^), and the HFPBS group was treated with combined probiotic-fermented milk containing a mixture of milks fermented with *L. rhamnosus* LV108, *L. casei* grx12, and *L. fermentum* grx08 (total viable count > 1 × 10^10^ cfu⋅mL^-1^). The animal groups and treatments are shown in Table 1.

**Table 1:** Animal groups and treatments.

Group	Diet	Gavage
Control (C)	Normal diet	Normal saline
High fat (HF)	High-fat diet	Normal saline
High fat with probiotics (HFPB)	High-fat diet	*L. rhamnosus* LV108 Fermented milk
High fat with probiotics (HFPBS)	High-fat diet	Mixture consisting of milk fermented with *L. rhamnosus* LV108, *L. casei* grx12, and *L. fermentum* grx08

### Measurement of Physiological and Biochemical Indexes

#### Weight Measurement in the Rats

The rats were weighed regularly each week, and the changes in weight were recorded. The dose administered via the gavage feeding was adjusted according to the body weight of the rats.

#### Measurement of the Rat Viscera Index

After 4 weeks of gavage feeding, the rats were weighed. The spleen, liver, and heart were removed, and the surrounding tissues were cleaned and weighed.

#### Serum and Viscera Biochemical Analysis

The total triglycerides (TGs), total cholesterol (TC), total bile acids (TBAs), high-density lipoprotein cholesterol (HDL-C), and low-density lipoprotein cholesterol (LDL-C) were determined using a Hitachi BS-7020 Automated Chemistry Analyzer (Hitachi Limited, Japan) using the corresponding TG, TC, TBA, HDL-C, and LDL-C kits purchased from the Nanjing Jiancheng Bioengineering Institute (Nanjing, Jiangsu, China). The very low-density lipoprotein cholesterol (VLDL-C) was determined using ELISA kits purchased from Shanghai Hualan Chemical Technology Co., Ltd. (Shanghai, China).

#### Histopathological Examination

The liver was removed, rinsed with physiological saline solution, blotted dry with filter paper, and weighed. The pathological lesions were observed, and the relative liver weights were calculated. The liver samples were immersion fixed, stored in 4% poly-formaldehyde solution, and routinely processed by embedding in paraffin. Five-micrometer sections were cut and stained with hematoxylin and eosin (H&E). The preparation of the tissue for the histopathology examination was conducted according to the methods described by Zhang et al. (2012).

### Real-Time Quantitative PCR (qPCR) Analysis of the Transcription of Genes

The total RNA from the liver and small intestine was isolated using an UNlQ-10 Column TRIzol Total RNA Isolation Kit (Sangon Biotech Co., Ltd., Shanghai, China) according to the manufacturer’s protocol. The tissue samples from each single group were pooled for the RNA extraction. The primers used for the reference gene (β-actin) and target genes were designed using the Primer 5.0 online software and are shown in Table 2. Quantitative real-time PCR was performed using an ABI Prism 7300 Detection System (Applied Biosystems, United States). All reactions were performed in duplicate using a kit provided by TaKaRa Biotechnology Company.

**Table 2:** List of genes measured in liver and small intestine tissues isolated from rats.

	Primer sequences (5′–3′)
LXRα(F)	CAGAGCCTACAGAACTTCGT
LXRα(R)	CTCGCAGCTCAGCACATT
LXRβ(F)	AAGCTGGTGAGCCTGCGC
LXRβ(R)	CGGCAGCTTCTTGTCCTG
ABCA1(F)	GGTGGTGTTCTTCCTCGTTAC
ABCA1(R)	GCTTCCGCTTCCTTCTGTAG
ABCG1(F)	GCCTGGCCATTGCACTAGAAC
ABCG1(R)	GGACACCACTTGGAAGCAAGA
ABCG5(F)	CGCAGGAACCGCATTGTAA
ABCG5(R)	TGTCGAAGTGGTGGAAGAGCT
SREBP-1C(F)	GCTGATGGAGACAGGGAGTT
SREBP-1C(R)	GCAGTTGATGTAGAGGCTAAGC
ChREBP(F)	CGAGGTGGTGATGCGTGAAT
ChREBP(R)	GAAGTTTGAAGATGTGGGCGT
AMPK(F)	CAGGCACATGGTTGTCCACAG
AMPK(R)	AATTTGGCGATCCACAGCTAGTTC
Hmgr(F)	CAACATCGTCACTGCCATC
Hmgr(R)	GATGCTCAAGCTGCCTTCT
SCD1(F)	TGCTGATGTGCTTCATCCTG
SCD1(R)	GGGAAACCAGGATATTCTCC
FAS(F)	GCCTTGCGTCACTTCCAGTTA
FAS(R)	GCTGAATACGACCACGCACTA
ACCα(F)	TGTCCTGCCCACTTTCTTCTAT
ACCα(R)	TCTTGCTGTCCTCCTCTGAGTA
PPARα(F)	TCAGTACATGTCTCTGTAGA
PPARα(R)	GGTCAGGGCCCGGGTCATACTCGCGGG
HMG-CoA(F)	GGTGGTGGGACCAACCTTCT
HMG-CoA(R)	CACGCCCCTTGAACACCTA
CYP7A1(F)	GGTTCTTCAGGTGTGAAACT
CYP7A1(R)	CAGAGATCTTGCCTGGCTCT
FXR(F)	TCCGAAGAAGCATCACCAAA
FXR(R)	CAGCCAACATTCCCATCTCTC
β-actin(F)	CCACTGGCATCGTGATGGAC
β-actin(R)	GCGGATGTCCACGTCACACT

The relative gene mRNA levels were determined using the Δcycle threshold (ΔCt) method, and β-actin served as a reference gene. For each target gene, the ΔΔCt values of all samples were calculated by subtracting the average ΔCt of the C group from the average ∆Ct of the HF, HFPB, or HFPBS group. The ΔΔCt values were converted to fold differences by raising 2 to the power of -ΔΔCt (i.e., 2^-ΔΔCT^).

### Statistical Analysis

All results are expressed as the mean ± standard deviation (SD). The differences among the groups were determined using a one-way ANOVA, and significant differences were considered significant at *P* < 0.05. SPSS software version 21.0 was used for all analyses.

## Results

### Body Weight and Organ Indexes

The weights of the rats and organs after dissection were determined to calculate the organ indexes. The final weights of the HF, HFPB, and HFPBS groups were significantly higher than the final weight of group C (*P* < 0.05, Table 3). The maximum increase in weight after 4 weeks was 225.34 g and occurred in the HF group, while the minimum value was 208.05 g and occurred in the HFPB group. The liver index values in the HF, HFPB, and HFPBS groups were significantly higher than those in the C group (*P* < 0.05, Table 3), but there were no significant differences in the other organ indexes.

**Table 3:** Body weight and organ indexes.

	Initial weight (g)	Final weight (g)	Cardiac index	Liver index	Spleen index	Renal index
C	141.65 ± 11.97^a^	332.02 ± 7.49^a^	0.32 ± 0.02^a^	2.53 ± 0.07^a^	0.17 ± 0.01^a^	0.54 ± 0.03^a^
HF	142.77 ± 16.01^a^	368.11 ± 10.05^b^	0.33 ± 0.04^a^	3.41 ± 0.09^b^	0.15 ± 0.01^a^	0.50 ± 0.07^a^
HFPB	138.36 ± 15.06^a^	346.41 ± 10.08^c^	0.30 ± 0.03^a^	3.24 ± 0.14^b^	0.15 ± 0.01^a^	0.51 ± 0.03^a^
HFPBS	144.53 ± 16.87^a^	362.94 ± 9.88^b^	0.33 ± 0.07^a^	3.27 ± 0.15^b^	0.15 ± 0.01^a^	0.52 ± 0.07^a^

*Values are expressed as the mean ± SD (*n* = 10). Different letters indicate significant differences among the groups (*P* < 0.05).*

### Lipid Profiles

The physiological and biochemical indexes in the serum, liver, and small intestine of the rats were quantified. In the serum, the TC, TG, VLDL-C, LDL-C, and TBA values in the HF group were significantly higher than those in the C group (*P* < 0.05, Table 4). After the probiotic intervention, the TC, TG, VLDL-C, LDL-C, and TBA values in the HFPB and HFPBS groups were significantly lower than those in the HF group (*P* < 0.05, Table 4), while the TC, TG, and VLDL-C values in the HFPB group were similar to those in the C group. The HFPB and HFPBS groups did not significantly differ in any of the indexes, except for TC, and the HFPBS group had a significantly higher TC value than the HFPB group (*P* < 0.05, Table 4).

**Table 4:** Serum physiological and biochemical indexes.

	TC	TG	VLDL-C	HDL-C	LDL-C	TBA
C	1.86 ± 0.07^a^	1.49 ± 0.17^a^	1.80 ± 0.04^a^	0.65 ± 0.5^a^	0.03 ± 0.02^a^	31.07 ± 0.19^a^
HF	2.73 ± 0.18^b^	1.95 ± 0.10^b^	2.39 ± 0.06^b^	0.50 ± 0.01^b^	0.14 ± 0.02^b^	49.43 ± 0.17^b^
HFPB	1.97 ± 0.17^a^	1.53 ± 0.21^a^	1.94 ± 0.10^ac^	0.52 ± 0.03^b^	0.06 ± 0.03^ad^	34.68 ± 0.87^c^
HFPBS	2.11 ± 0.12^c^	1.62 ± 0.05^a^	2.07 ± 0.11^c^	0.51 ± 0.03^b^	0.09 ± 0.04^d^	38.13 ± 0.33^c^

*Values are expressed as the mean ± SD (*n* = 10). Different letters indicate significant differences among the groups (*P* < 0.05).*

In the liver, the TC, TG, and TBA values in the HF group were significantly higher than those in the C group (*P* < 0.05, Table 5). After the probiotic intervention, the values of TC, TG, and TBA in the HFPB and HFPBS group were significantly lower than those in the HF group (*P* < 0.05, Table 5), and the values of TG were similar to those in the C group. The value of TC in the HFPB group was significantly lower than that in the HFPBS group (*P* < 0.05, Table 5).

**Table 5:** Liver physiological and biochemical indexes.

	TC	TG	TBA
C	0.025 ± 0.004^a^	0.098 ± 0.016^a^	1.556 ± 0.024^a^
HF	0.065 ± 0.007^b^	0.134 ± 0.017^b^	1.740 ± 0.017^b^
HFPB	0.043 ± 0.002^c^	0.100 ± 0.007^a^	2.020 ± 0.020^c^
HFPBS	0.043 ± 0.007^c^	0.090 ± 0.013^a^	1.966 ± 0.024^c^

*Values are expressed as the mean ± SD (*n* = 10). Different letters indicate significant differences among the groups (*P* < 0.05).*

In the small intestine, the TC, TG, and TBA values in the HF group were significantly higher than those in the C group (*P* < 0.05, Table 6). After the probiotic intervention, the values of TC, TG, and TBA in the HFPB and HFPBS groups were significantly lower than those in the HF group (*P* < 0.05, Table 6), and the values of TC were similar to those in the C group (*P* > 0.05, Table 6). The TC and TBA values in the HFPBS group were significantly higher than those in the HFPB group (*P* < 0.05, Table 6).

**Table 6:** Small intestine physiological and biochemical indexes.

	TC	TG	TBA
C	0.051 ± 0.009^a^	0.181 ± 0.072^a^	1.270 ± 0.266^a^
HF	0.077 ± 0.019^b^	0.308 ± 0.034^b^	1.480 ± 0.183^b^
HFPB	0.052 ± 0.010^a^	0.237 ± 0.011^c^	1.690 ± 0.262^c^
HFPBS	0.059 ± 0.006^a^	0.272 ± 0.013^d^	1.790 ± 0.259^d^

*Values are expressed as the mean ± SD (*n* = 10). Different letters indicate significant differences among the groups (*P* < 0.05).*

### Histopathological Examination

**FIGURE 1:**
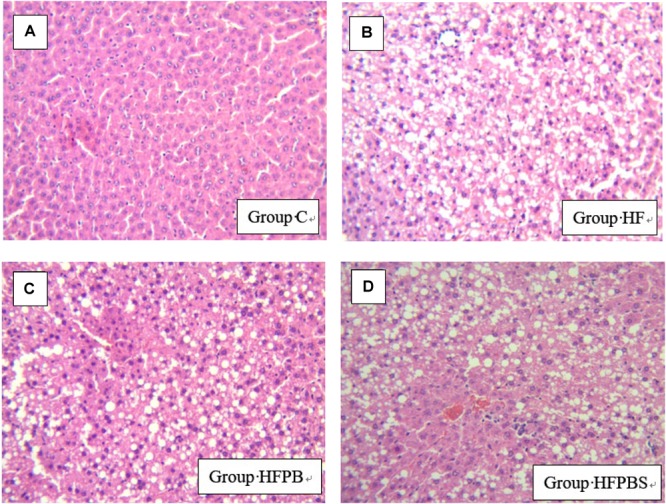
Histopathological examination of liver tissue showing damage and arrangement of hepatocytes and fat vacuoles (100× magnification) (*n* = 10). **(A)** Tissue from the C group showing a normal liver with no lipid deposits. **(B)** Liver tissue from the HF group showing the presence of steatosis. **(C)** Tissue from the HFPB group showing the presence of steatosis. **(D)** Cells from the HFPBS group containing many large fat vacuoles.

The liver in the C group (Figure 1A) was normal. The hepatic sinusoids were clearly visible, and the hepatic cord was regularly arranged. However, diffuse lipid changes were found in the HF group (Figure 1B). The liver cells were enlarged; the hepatic sinusoids were narrowed or even disappeared; the structure of the hepatic cord was disorganized; and some manifestations of hepatocyte edema, punctate necrosis, and infiltration of inflammatory cells were observed. The liver injury in the HFPB and HFPBS groups (Figure 1C,D, respectively) was reduced, the number of fatty liver cells was decreased, and the number of lipid droplets in the cytoplasm was also decreased sometimes to zero.

### Transcription Levels of Genes Associated With Lipid Metabolism in Rat Livers

**FIGURE 2:**
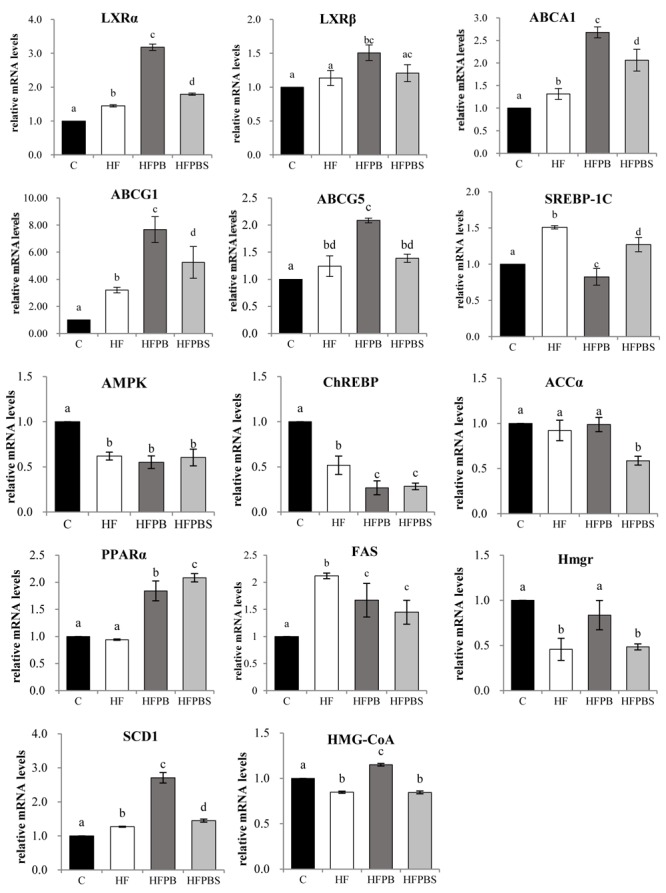
Effects of diet on the transcription of genes related to lipid metabolism in freshly isolated livers. The total RNA was extracted, reverse transcribed to cDNA, and analyzed to detect the transcription of genes using RT-PCR. Gene transcription is reported as the mean ± SD (*n* = 6) of the fold change relative to the C group. Error bars with different letters indicate significant differences (*P* < 0.05).

The transcription levels of the genes associated with lipid metabolism in rat livers are illustrated in Figure 2. In the HF group, the transcription of genes involved in the regulation of adipogenesis (SREBP-1C, FAS, and SCD1) and cholesterol transport and metabolism (LXRα, LXRβ, ABCA1, ABCG1, and ABCG5) was significantly increased compared to that in the C group (*P* < 0.05, Figure 2), while the transcription of genes involved in fatty acid β-oxidation (PPARα and ChREBP) was significantly decreased.

Upregulated LXRα, LXRβ, ABCA1, ABCG1, SCD1, and PPARα gene transcription levels and downregulated SREBP-1C and FAS gene transcription levels were observed in the HFPB and HFPBS groups relative to those in the HF groups (*P* < 0.05, Figure 2). Furthermore, the ABCG5, Hmgr, and HMG-CoA gene transcription in the HFPB group were significantly higher than that in the HF group (*P* < 0.05, Figure 2).

However, in the HFPB group, the transcription of genes involved in the regulation of cholesterol transport and metabolism (LXRα, LXRβ, ABCA1, ABCG1, ABCG5, Hmgr, and HMG-CoA) was significantly increased compared to that in the HFPBS group (*P* < 0.05, Figure 2). Furthermore, the SREBP-1C and PPARα gene transcription levels in the HFPB group were significantly higher than that in the HFPBS group (*P* < 0.05, Figure 2).

### Transcription Levels of Genes Associated With Lipid Metabolism in the Small Intestine of Rats

**FIGURE 3:**
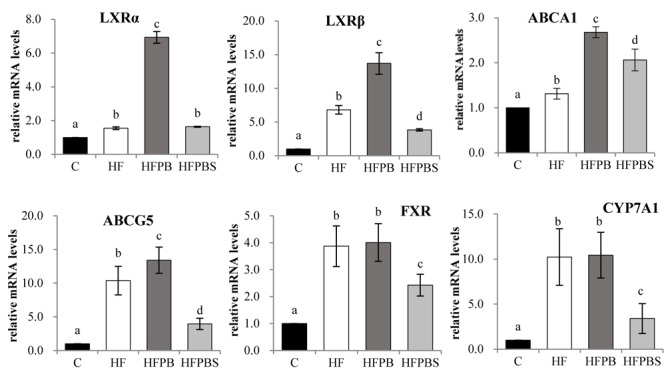
Effects of diet on the transcription of genes related to lipid metabolism in the small intestine. The total RNA was extracted from freshly isolated small intestines, reverse transcribed to cDNA, and analyzed to detect the transcription of genes using RT-PCR. Gene transcription is reported as the mean ± SD (*n* = 6) of the fold change relative to the C group. Error bars with different letters indicate significant differences (*P* < 0.05).

The transcription levels of the genes associated with lipid metabolism in the small intestine of rats are illustrated in Figure 3. Compared to the C group, feeding the rats a high-fat diet significantly increased the transcription of genes involved in cholesterol transport and metabolism (LXRα, LXRβ, ABCA1, and ABCA5) (*P* < 0.05, Figure 3).

In the HFPB group, the transcription of genes involved in the regulation of adipogenesis (SCD1 and PPARα) and cholesterol transport, and the metabolism (LXRα, LXRβ, ABCA1, and ABCA5) was significantly increased compared with that in the HF group (*P* < 0.05, Figure 3).

In the HFPBS group, the transcription of genes involved in cholesterol transport and metabolism (ABCA1) was significantly increased, and the transcription of LXRβ, ABCA5, FXR, and CYP7A1 was decreased compared with that in the HF group (*P* < 0.05, Figure 3). Furthermore, transcription in the HFPBS group was significantly lower than that in the HFPB group (*P* < 0.05, Figure 3).

## Discussion

Based on previous studies, this study attempts to analyze the multifaceted mechanism by which probiotics reduce lipid levels in their hosts by evaluating the transcription of the genes involved in the LXR, AMPK, and FXR axes of lipid metabolism. In addition, the effects of single and combined probiotic-fermented dairy products on the lipid level and transcription of lipid metabolism-related genes in hyperlipidemic rats were compared. The results show that probiotic-fermented dairy products significantly reduced the levels of TC and TG in the serum, liver, and small intestine of the rats and reduced lipid accumulation in the liver. There were no significant differences between the single and combined probiotic-fermented dairy products. Other reports have shown that *L. acidophilus, L. plantarum* 9-41-A, *L. fermentum* M1-16, and *L. plantarum* MA2 are able to reduce the liver TC and lipid levels (Fukushima and Nakano, 1996; Wang et al., 2009; Xie et al., 2011), while another study also showed that the probiotic *L. gasseri* SBT2055 can reduce not only abdominal visceral fat but also subcutaneous fat (Kadooka et al., 2010).

Compared to the HF group, the transcription of genes involved in the LXR axis in the liver and small intestine was significantly upregulated in both the HFPB and HFPBS groups. LXRs are the main “receptors” maintaining the relative stability of intracellular cholesterol. The LXRs predominantly maintain cholesterol balance through the following processes: (1) inhibition of the intestinal absorption of cholesterol in food; (2) regulation of the synthesis and absorption of cholesterol in the liver; (3) regulation of the reverse transport of cholesterol; and (4) promotion of the transformation of cholesterol to bile acid and cholesterol excretion through bile (Trasino et al., 2013). The transcription of LXRα, LXRβ, ABCA1, ABCG1, and ABCG5 in the liver and small intestine in the HFPB and HFPBS group was significantly higher than that in the HF group (*P* < 0.05). The transcription of the LXR genes in the liver and small intestine of the rats was considered activated by the probiotics, thus upregulating the transcription of genes involved in the LXR axis. The upregulated transcription of ABCA1 and ABCG1 increased cellular cholesterol efflux to HDL-C in the serum, which increased RCT. The upregulation of ABCG5 promotes the secretion and excretion of cholesterol in the hepatobiliary tube. Furthermore, upon activation, the LXR in the small intestine has been observed to be activated, and the transcription of NPC1 L1 was downregulated (Wang et al., 2006), which reduced the absorption of cholesterol, increased the transcription of ABCG5, and mediated the flow of sterols in intestinal cells to the absorption of cholesterol in the intestinal cavity (Wang et al., 2006). Therefore, the content of TC in the serum and liver in the HFPB and HFPBS groups (Table 4) was significantly lower than that in the HF group (*P* < 0.05), while the content of TBA was significantly increased (*P* < 0.05). In addition, in the intestinal tract, the upregulation of the FXR and CYP7A1 genes as a result of the administration of the probiotics-fermented milks was not significantly compared with that in the HF group, and that in the HFPBS group was significantly lower than that in the HF group. Thus, it is possible that a decrease in the host TC caused by probiotics may not be achieved by affecting the genes involved in the FXR axis.

Regarding the transcription of triglyceride-related genes in the liver and small intestine, there were no significant differences in AMPK transcription between the HFPB and HFPBS groups compared with the HF group, but the transcription of LXRα, PPARα, and SCD-1 in the liver was significantly upregulated, while the transcription of ChREBP and FAS was significantly downregulated (*P* < 0.05). First, ChREBP is a target gene of LXRs and can upregulate the transcription of liver-type pyruvate kinase. ChREBP and liver-type pyruvate kinase play important roles in the conversion of carbohydrates to lipids. A study by Uyeda et al. (2002) showed that LXR agonists can promote the conversion of carbohydrates to fat in the liver, leading to the accumulation of fat in this organ. However, in the current study, the probiotics activated the transcription of the LXR gene but reduced the transcription of the ChREBP gene, which inhibited the transformation of carbohydrates into fat in the liver and reduced hepatic fat accumulation. Similarly, the treatment with the probiotics resulted in an upregulation of the SCD-1 gene in the liver. SCD-1 reduces free saturated fatty acids in liver cells, thus reducing the induction of lipid peroxidation in liver cells. Second, the treatment with the probiotics resulted in the upregulation of the LXRα and PPARα genes in the liver, and a study by Trasino et al. (2013) showed that LXRα can inhibit the differentiation of mesenchymal stem cells into adipocytes through Wnt/β-catenin (β-catenin) signaling. PPARα regulates peroxidase, fatty acid transport, and mitochondrial fatty acid β-oxidation metabolism gene transcription and expression, thereby promoting the decomposition, absorption, and utilization of fatty acids (Zhou et al., 2008). Therefore, the treatment with the probiotics did not directly activate AMPK, but the level of TG in the rat serum, liver, and small intestine was significantly reduced by the activation of the transcription of PPARα and LXRα (*P* < 0.05).

Notably, the effects of the probiotic intake showed the same trend in both the rat liver and small intestine in the rats fed the HF diet only compared to the rats in the C group (normal diet), i.e., increased transcription of most genes. The possible reasons for these results are as follows. First, the daily diet was an important factor affecting lipid metabolism. When a large amount of fat is provided in the diet, the transcription levels of the genes maintaining homeostasis in lipid metabolism (e.g., LXRs, AMPK, and FXR) were also upregulated to maintain the lipid levels. The HF, HFPB, and HFPBS groups were fed a high-fat diet; thus, these groups showed similar trends. However, after the probiotics administration intervention, the lipid metabolism disorder induced by the high-fat diet was alleviated, further indicating that the administration of the probiotics was effective. Second, the degree of hepatocytes steatosis that the histopathological examination showed was not sufficient to stop lipid metabolism, so rats could still undergo feedback regulation when they ingested a large quantity of lipids. In addition, compared with the HF group, hepatocytes steatosis was obviously alleviated in the HFPB and HFPBS group, further suggesting that the damage was reversible and that the probiotics administration intervention was effective.

Furthermore, there were no significant differences in the physiological and biochemical indexes between the HFPB and HFPBS groups, except TC level, and the ability of the rats in the HFPB group to regulate the transcription of cholesterol metabolism-related genes was significantly better than that in those in the HFPBS group (*P* > 0.05). Notably, LXR agonists can cause the accumulation of liver fat and an increase in TG levels in the plasma, which mainly occurs because LXRs induce the transcription of SREBP1c. SREBP1c is a major transcription factor of the fat synthesis gene, which activates a variety of enzymes involved in fatty acid and triglyceride synthesis. Therefore, researchers believe that new LXR agonists can regulate some repressor and auxiliary activators to selectively upregulate ABCA1 without upregulating the transcription of SREBP1c (Beyea et al., 2007). The transcription of SREBP1c was significantly downregulated in the HFPB group but significantly upregulated in the HFPBS group. Therefore, we believe that the long-term use of *L. rhamnosus* LV108-fermented dairy products has a better effect on reducing lipid accumulation in the liver, small intestine, and blood.

## Conclusion

The milk fermented with a single probiotic (*L. rhamnosus* LV108) and the combined probiotic (*L. rhamnosus* LV108, *L. casei* grx12, and *L. fermentum* grx08)-fermented milk significantly improved the lipid levels and visceral lipid accumulation in hyperlipidemic rats. At the physiological and biochemical levels, there were no significant differences between the single probiotic- and combined probiotic-fermented milks in the hyperlipidemic rats; however, the regulation of the transcription of lipid metabolism-related genes caused by the single probiotic-fermented milk was more effective than that by the combined probiotic-fermented milk.

## Ethics Statement

This study was carried out in accordance with the recommendations of the China Institutional and National Guidelines, Ethics Committee of the Yangzhou University. The protocol was approved by the Ethics Committee of the Yangzhou University.

## Author Contributions

YW, BY, and RG participated in the design of this study. YW and BY contributed equally to this work and performed the statistical analysis. YoH, WX, YiH, CW, and FG carried out the study and collected important background information. YW drafted the manuscript. All authors read and approved the final manuscript.

## Conflict of Interest Statement

The authors declare that the research was conducted in the absence of any commercial or financial relationships that could be construed as a potential conflict of interest.
